# Photon counting detector CT contrast agent-reduced transcatheter aortic valve reconstruction planning: a comparative study

**DOI:** 10.1007/s00330-026-12613-5

**Published:** 2026-05-06

**Authors:** Yannik C. Layer, Alexander Isaak, Narine Mesropyan, Patrick A. Kupczyk, Dmitrij Kravchenko, Marilia Voigt, Tatjana Dell, Julian A. Luetkens, Daniel Kuetting

**Affiliations:** Department of Diagnostic and Interventional Radiology, University Hospital Bonn, Venusberg-Campus 1, 53127 Bonn, Germany

**Keywords:** Contrast media, Photon-counting detector computed tomography, Dose reduction, Iodine signal, TAVR planning

## Abstract

**Objectives:**

Continuous efforts are made to reduce contrast media, improving patient safety, reducing environmental risks, and addressing recurring supply shortages. The aim of this study was to evaluate contrast agent-reduced CT protocols for transcatheter aortic valve reconstruction (TAVR) planning in photon counting detector CT (PCDCT).

**Materials and methods:**

162 BMI-matched examinations with standard dose contrast media (SCD; 80 mL; Iohexol 300 mg/mL; 81 examinations) and reduced contrast media dose (RCD; 50 mL; 81 examinations) for TAVR planning on a PCDCT were included in this retrospective monocentric study. Virtual monoenergetic reconstructions (VMI) at 70 keV, 60 keV and 50 keV of contrast agent-reduced examinations were compared with polyenergetic images. Quantitatively, regions-of-interest (ROIs) were placed in the abdominal aorta, iliac bifurcation, femoral artery, left ventricle and trapezius muscles. Signal-to-noise-ratio (SNR) and contrast-to-noise-ratio (CNR) were calculated. Qualitatively, diagnostic quality and contrast were assessed on a visual grading scale of 1 (non-diagnostic) – 5 (excellent) and contrast agent dose was estimated.

**Results:**

Averaged, SNR and CNR decreased by 8.71% and 16.78%, respectively, on PCDCT with reduced contrast dose (RCD vs. SCD; both *p* < 0.001). VMI50keV increased SNR by 44.10% (*p* < 0.001) and CNR by 52.73% (*p* < 0.001) compared with SCD. In the ascending aorta, SNR increased from 19.80 ± 6.24 (SCD) to 35.78 ± 13.20 (RCD_VMI50keV_) and CNR from 18.84 ± 7.78 to 29.77 ± 16.70. Median contrast intensity was 5 for SCD, 4 for RCD_CR_, and 5 for RCD_VMI50keV_.

**Conclusion:**

The diagnostic efficacy of TAVR planning assessment using PCDCT with minimized contrast agent dosing is preserved, presenting a practical approach to conserve contrast media.

**Key Points:**

***Question*** *The aim of the study was to implement a PCDCT-adapted contrast media dose protocol to reduce contrast agent volume at sufficient diagnostic quality.*

***Findings*** *PCDCT enables substantial contrast dose reduction for TAVR planning with maintained diagnostic image quality. Low-keV virtual monoenergetic image reconstructions compensate for the reduced iodine concentration.*

***Clinical relevance*** *The study demonstrates the potential of contrast media reduction of PCD-CT in clinical routine. This can benefit patients with renal impairment, for example, and reduce the negative effects of iodinated contrast media on the environment.*

**Graphical Abstract:**

**Figure:**
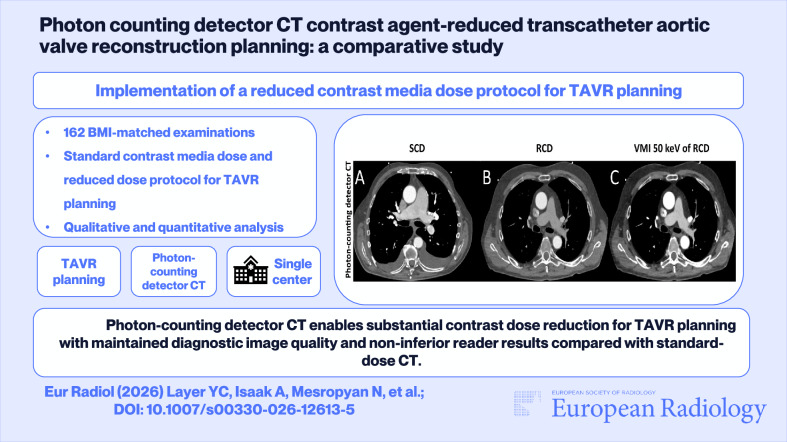

## Introduction

Transcatheter aortic valve reconstruction (TAVR) has become the standard therapy, especially for elderly and often frail patients with severe aortic valve stenosis [1]. Pre-procedural CT is the cornerstone of TAVR planning, as it provides a detailed assessment of vascular access routes and precise measurement of aortic root anatomy [2]. Although there are data supporting non-contrast CT approaches, the administration of iodinated contrast media remains a requisite for reliable CT-based TAVR planning [3–5]. This patient cohort frequently presents with impaired renal function, making contrast administration a relevant clinical concern [6, 7]. While the risk of contrast-associated nephropathy has been re-evaluated and somewhat downgraded in recent years, TAVR patients are still considered at increased risk for contrast-associated acute kidney injury [8–12]. Beyond patient safety, additional factors favor contrast media reduction—including the potential environmental impact of contrast residues in groundwater [13], medical considerations such as hyperthyroidism or anaphylactic reactions, financial implications, and the experience of contrast shortages during the COVID-19 pandemic [14, 15].

A well-established strategy for the reduction of contrast media volume is low kV image acquisition, exploiting the fact that iodine attenuation increases as the X-ray spectrum approaches the iodine k-edge of 33 keV [16]. Similarly, dual-energy CT allows the reconstruction of low-energy virtual monoenergetic images (VMIs) that enhance iodine signal and can therefore enable a lower contrast dose [17–19]. Consequently, many publications recommend additional reconstruction of VMI in routine cardiovascular imaging [14, 17, 20–23]. Photon-counting detector CT enables the direct conversion of photons into electrical signals, resulting in more sensitive and accurate photon detection compared with conventional energy-integrating detector CT. This enables spectral resolution and offers improved iodine sensitivity [24]. The technology, therefore, holds considerable potential for reducing contrast media requirements [14, 25, 26].

Accordingly, our aim was to implement a reduced contrast media dose (RCD) protocol to minimize contrast agent volume at sufficient diagnostic quality in PCDCT and evaluate its diagnostic performance for TAVR planning.

## Materials and methods

In a single-center, retrospective study design, patients receiving a CT for TAVR planning between January 2022 and September 2023 on a photon-counting detector CT (NAEOTOM Alpha, Siemens Healthcare GmbH, VA50) were included in this study. Patients of the differing contrast media protocols were matched for Body Mass Index.

The study was approved by the local ethics review board (Ethics Committee of the Medical Faculty of Bonn; file reference 027/23). The need for informed consent was waived. Scans were acquired during clinical routine. The study is in compliance with the Declaration of Helsinki and its amendments.

### Imaging protocol

All patients received the same imaging protocol. The following scan parameters were used: ECG-gated scan at 60% of the R-R interval; tube voltage of 120 kVp with activated automatic tube current modulation; pitch of 3.2. CARE keV was set to 64. Collimation was 144 ×0.4 mm. Slice thickness of the reconstruction was set to 1 mm, and an increment of 0.8 mm was employed. Images were reconstructed on a 512 × 512 pixel grid, with a pixel size of 0.72 mm. For image reconstruction, a regular quantitative kernel (Qr40; Siemens Healthcare GmbH) and Quantum Iterative Reconstruction (QIR Level 4; Siemens Healthcare GmbH) were used. Patients were placed in a head-first supine position.

### Contrast injection protocol

For intravenous administration of iodine-based contrast agent (Iohexol, Accupaque 300 mg/mL, GE Healthcare Buchler GmbH & Co. KG), the SCD group patients received a bolus of 80ml of contrast media, followed by a 60 mL bolus of physiologic saline solution, both at a flow rate of 4.5 mL/s.

In the RCD group, a triphasic bolus technique was used. Patients received a bolus of 50 mL mixture of 85% contrast media and 15% physiologic saline solution at a flow rate of 3 mL/s, followed by 40 ml of mixture of 25% contrast media and 75% physiologic saline solution at a flow rate of 4.5 mL/s. 30 mL of sole physiologic saline solution was administered afterward at a flow rate of 4.0 mL/s. This led to a total administration of 52.5 mL of contrast media in the RCD group.

Bolus tracking was performed by placing a circular region of interest (ROI) in the descending aorta. The scan was triggered once the attenuation within the ROI reached 120 Hounsfield Units (HUs), followed by a post-threshold delay of 5 seconds. For both SCD and RCD groups, conventional reconstructed polyenergetic images (CR) were reconstructed. For the RCD group, VMI of RCD at 70, 60, and 50 keV were reconstructed based on spectral post-processing (SPP) images. This was done using the vendor’s regular software (Syngo.Via VB70, Siemens Healthcare GmbH) with the “MM Reading” application. The VMI level was adjusted as required and uploaded to the PACS system.

### Quantitative image analysis

ROIs were placed in the following regions: Ascending aorta, abdominal aorta, above the iliac bifurcation, left and right common femoral artery, left ventricle, and the left and right trapezius muscle for quantitative analysis. The ROI was selected to be as large as possible while carefully excluding lesions such as calcifications, stents, artifacts, or other adjacent structures that could affect attenuation measurements. Placement of ROIs was conducted using a regular clinical DICOM viewer (Deep Unity R20 XX; Dedalus HealthCare GmbH). Values and standard deviation of attenuation were evaluated and averaged for matched measurements (e.g., right and left common femoral artery). Based on this, signal-to-noise ratio (SNR = HU target/standard deviation target (SDtarget)) and contrast-to-noise ratio (CNR = (HUtarget – HUmuscle/SDmuscle) were calculated. Image noise was defined as the standard deviation of the measured CT numbers.

Additionally, the aortic annulus diameter was measured using a regular clinical DICOM viewer. Aortic annulus measurements were performed using multiplanar reconstruction (MPR) centered on the annulus.

### Qualitative image analysis

Qualitative assessment was evaluated by two radiologists with three (Y.C.L.) and six (A.I.) years of experience in CT imaging. Both raters assessed CT images regarding contrast intensity and diagnostic image quality using a five-point visual grading scale for each criterion. The rating was defined as follows: (1) non-diagnostic; (2) poor, insufficient diagnostic confidence; (3) moderate, low diagnostic confidence; (4) good, diagnostic with high confidence; and (5) excellent, full diagnostic confidence of the vessels. Additionally, readers categorized examinations as either regular or reduced contrast agent quantity. The readers were blinded to technical data and rated the acquired images in random order.

### Statistical analysis

For statistical analyses, IBM SPSS Version 27 (IBM Corp.) was used. Graphs were plotted using GraphPad PRISM Version 6.02 (GraphPad Software). Quantitatively, results are expressed as mean and standard deviation. Shapiro–Wilk test, Mann-Whitney test and unpaired *t*-test were used for statistical analysis of qualitative and quantitative image parameters as appropriate. Qualitative results are stated as median with interquartile range (IQR). The intraclass correlation coefficient (ICC) was used for the assessment of interrater reliability. ICC estimates, and their 95% confident intervals (CI) were calculated on the basis of a mean-rating (*k* = 2), consistency, two-way mixed-effects model. *p*-values below 0.01 were defined as significant.

## Results

### Participant characteristics

Overall, 111 male and 51 female patients were included in the analysis, resulting in a median age of 79 (72–83) years. Median age was 78 (70.5–83) years for SCD and 80 (74–83.5) years for RCD, respectively. Patients were matched one-to-one based on their BMI. Mean BMI was 26.66 kg/m² for SCD and 26.62 kg/m² for RCD (*p* < 0.01). 20 patients were considered obese (BMI > 30) and 12 were highly obese (BMI > 35). Mean dose length product (DLP) for SCD was 263.71 mGy*cm (RCD: 279.21 mGy*cm; *p* = 0.09) and mean CTDI_vol_ was 3.75 mGy (RCD: 3.93 mGy; *p* < 0.10). Patient characteristics are shown in Table 1.

**Table 1:** Patient characteristics of the standard contrast media dosage group (SCD) and the reduced contrast media dosage group (RCD)

	SCD	RCD
*N*	81	81
Sex, female, *n*	24	27
Obese (BMI > 30), *n*	10	10
Highly obese (BMI > 35), *n*	6	6
Median age, years (range)	78 (70.5–83)	80 (74–83.5)
Mean BMI, kg/m²	26.66	26.62
Mean DLP, mGy*cm	263.71	279.21
Mean CTDI_vol_, mGy	3.75	3.93

### Quantitative image analysis

**Fig. 1:**
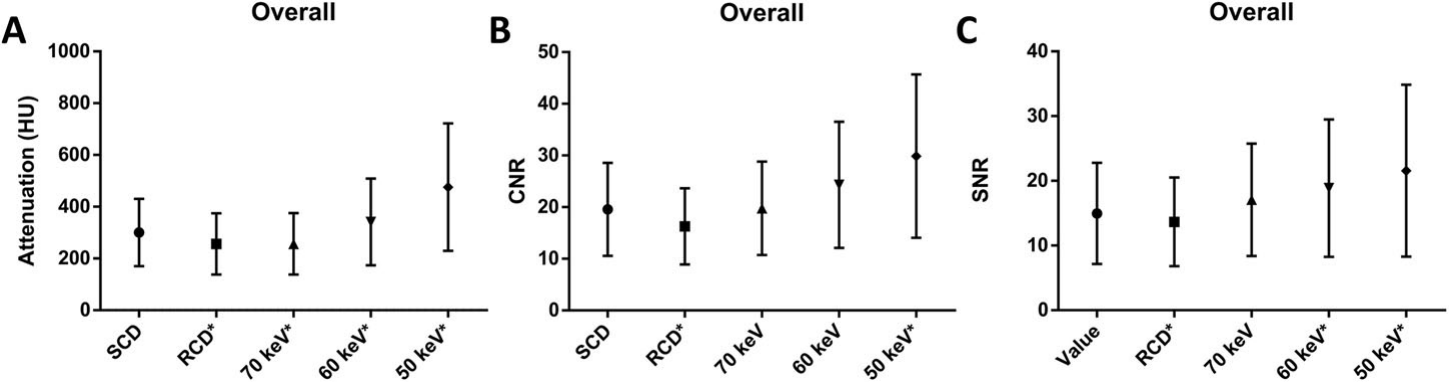
Mean overall attenuation (**A**), contrast-to-noise ratio (CNR) (**B**), and signal-to-noise ratio (SNR) (**C**) and corresponding standard deviation within defined regions of interest plotted for photon-counting detector CT (PCDCT) at standard contrast media dose (SCD), reduced contrast media dose (RCD), and virtual monoenergetic images (VMI) of RCD at 70, 60, and 50 keV. *p*-values below 0.01 are indicated with an asterisk

Results of quantitative image analysis are summarized in Tables 2, 3 and 4 and Fig. 1. RCD images demonstrated a mean decrease of 8.71% in SNR and 16.78% in CNR in the conventional polyenergetic reconstructions compared with SCD images (both *p* < 0.001). Reconstruction with VMI at 50 keV significantly improved attenuation (+ 58.87%), SNR (+ 44.10%), and CNR (+ 52.73%) relative to SCD (all *p* < 0.001). Similar improvements were observed throughout the aorto-iliofemoral axis; for example, in the ascending aorta, SNR increased from 19.80 ± 6.24 (SCD) to 35.78 ± 13.20 (RCD VMI50 keV), and CNR from 18.84 ± 7.78 to 29.77 ± 16.70. Deviation of image noise between RCD and SCD remained non-significant (RCD: 21.14; SCD: 19.76; *p* = 0.12). Aortic annulus diameter remained unchanged between RCD and VMI (Mean diameter: 24.1 ± 1.5 mm vs. 24.2 ± 1.5 mm; *p* = 0.912).

**Table 2:** Mean attenuation values and standard deviation within defined regions of interest for photon-counting detector CT (PCDCT) at standard contrast media dosage (SCD), reduced contrast media dosage (RCD), and RCD combined with virtual monoenergetic images (VMI) at 70, 60, and 50 keV

	*n*	Ascending aorta	Abdominal aorta	Iliac bifurcation	Left common femoral artery	Right common femoral artery	Left ventricle	Muscle
Standard	81	337.7 ± 18.0	362.4 ± 25.3	367.8 ± 24.7	351.8 ± 21.9	356.9 ± 20.3	305.2 ± 22.9	56.8 ± 16.0
Reduced	81	**292.9** ± **14.8**	325.6 ± 23.2	326.7 ± 22.4	**285.3** ± **22.4**	**288.8** ± **20.3**	**256.8** ± **19.4**	51.2 ± 15.6
VMI 70 keV	81	**292.8** ± **11.4**	326.0 ± 17.7	322.6 ± 17.7	**286.4** ± **19.7**	**291.6** ± **17.9**	**260.3** ± **15.5**	51.2 ± 13.0
VMI 60 keV	81	**391.4** ± **13.3**	442.9 ± 21.5	**438.8** ± **21.2**	379.6 ± 25.7	391.8 ± 22.7	341.9 ± 18.7	49.1 ± 15.4
VMI 50 keV	81	**551.0** ± **15.7**	**632.8** ± **26.1**	**627.2** ± **25.9**	**530.5** ± **34.9**	**536.8** ± **29.5**	**476.3** ± **23.1**	45.6 ± 19.1

Bold results were considered significant compared to SCD (*p* < 0.01)

**Table 3:** Mean signal-to-noise ratio (SNR) and standard deviation (SD) within defined regions of interest for photon-counting detector CT (PCDCT) at standard contrast media dosage (SCD), reduced contrast media dosage (RCD) and RCD combined with virtual monoenergetic images (VMI) at 70, 60, and 50 keV

	*n*	Ascending aorta	Abdominal aorta	Iliac bifurcation	Left common femoral artery	Right common femoral artery	Left ventricle	Muscle
Standard	81	19.8 ± 6.2	15.0 ± 4.7	15.8 ± 5.8	17.3 ± 7.4	19.4 ± 9.2	13.9 ± 4.7	3.8 ± 1.3
Reduced	81	20.0 ± 6.5	14.5 ± 4.8	15.2 ± 5.8	**14.0** ± **5.6**	**15.3** ± **5.7**	13.4 ± 3.9	3.4 ± 1.1
VMI 70 keV	81	**26.0** ± **8.4**	**19.1** ± **6.2**	**18.9** ± **5.8**	16.6 ± 7.5	17.9 ± 6.9	**17.0** ± **5.2**	4.2 ± 1.3
VMI 60 keV	81	**29.9** ± **10.5**	**21.5** ± **7.4**	**21.8** ± **7.3**	17.7 ± 9.6	19.2 ± 8.1	**18.6** ± **6.0**	3.4 ± 1.2
VMI 50 keV	81	**35.8** ± **13.2**	**25.4** ± **9.2**	**25.8** ± **9.3**	19.0 ± 11.3	21.0 ± 10.3	**21.3** ± **7.4**	2.6 ± 1.2

SNR was calculated as target attenuation values/target SD. Bold results were considered significant compared to SCD (*p* < 0.01)

**Table 4:** Mean contrast-to-noise ratio (CNR) and standard deviation (SD) within defined regions of interest for photon-counting detector CT (PCDCT) at standard contrast media dosage (SCD), reduced contrast media dosage (RCD) and RCD combined with virtual monoenergetic images (VMI) at 70, 60, and 50 keV

	*n*	Ascending aorta	Abdominal aorta	Iliac bifurcation	Left common femoral artery	Right common femoral artery	Left ventricle
Standard	81	18.8 ± 7.8	20.6 ± 8.6	20.9 ± 9.0	20.0 ± 9.9	20.3 ± 9.9	16.7 ± 8.1
Reduced	81	16.2 ± 7.7	18.2 ± 7.4	18.3 ± 7.5	**15.4** ± **6.7**	**15.7** ± **6.7**	13.8 ± 7.3
VMI 70 keV	81	19.6 ± 9.4	22.2 ± 9.1	21.9 ± 8.5	18.7 ± 8.2	19.2 ± 8.7	17.0 ± 9.5
VMI 60 keV	81	**24.1** ± **12.8**	**27.5** ± **12.7**	**27.1** ± **11.9**	22.7 ± 11.0	23.6 ± 11.7	20.7 ± 12.0
VMI 50 keV	81	**29.8** ± **16.7**	**34.2** ± **16.1**	**33.8** ± **16.0**	**27.9** ± **14.3**	**27.9** ± **14.1**	**25.6** ± **15.7**

CNR was calculated as (target attenuation − muscle attenuation)/muscle SD. Bold results were considered significant compared to SCD (*p* < 0.01)

### Qualitative image analysis

**Fig. 2:**
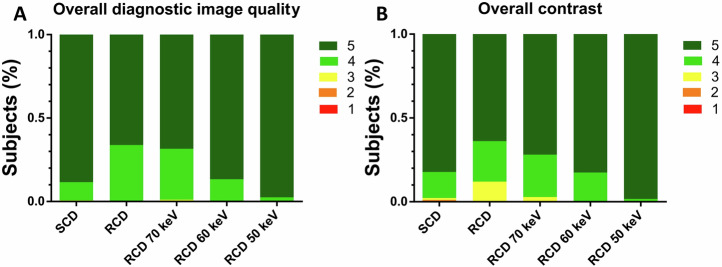
Bar plots show the distribution of overall contrast (**A**) and overall diagnostic quality rating (**B**) with standard contrast media dose (SCD), reduced contrast media dose (RCD), and virtual monoenergetic images (VMI) of RCD at 70, 60, and 50 keV

Results of qualitative image analysis are summarized in Table 5 and Fig. 2. Diagnostic image quality was preserved with RCD compared with SCD (median [IQR]: SCD 5 [5]; RCD CR 5 [4,5]; RCD VMI70 keV 5 [4,5]). Subjective contrast ratings were likewise non-inferior for RCD VMI70 keV relative to SCD (SCD 5 [5]; RCD CR 4 [4,5]; RCD VMI70 keV 5 [4,5]). In blinded assessment, 77.16% (125/162) of RCD examinations were classified as SCD, indicating preserved perceived contrast enhancement. Interrater agreement was excellent (overall ICC = 0.856; 95% CI: 0.818–0.8886), including ICC values of 0.812 (95% CI: 0.741–0.865) for diagnostic quality, 0.874 (95% CI: 0.823–0.910) for contrast, and 0.922 (95% CI: 0.889–0.941) for SCD classification.

**Table 5:** Median and inter-quartile range (IQR) of qualitative image ratings for images acquired at standard contrast media dosage (SCD), reduced contrast media dosage (RCD), and RCD combined with virtual monoenergetic images (VMI) at 70, 60, and 50 keV

	SCD	RCD	VMI 70 keV	VMI 60 keV	VMI 50 keV	ICC
Contrast intensity	5 (5-5)	4 (4-5)	5 (4-5)	5 (5-5)	5 (5-5)	0.874 (95% CI: 0.823–0.910)
Overall diagnostic quality	5 (5-5)	5 (4-5)	5 (4-5)	5 (5-5)	5 (5-5)	0.812 (95% CI: 0.741–0.865)
Classified SCD	95.68%	77.16%	96.58%	100%	100%	0.934 (95% CI: 0.899–0.961)

Intraclass correlation coefficient (ICC) estimates and their 95% confidence intervals (CIs) were calculated. ICC calculation is based on a mean-rating (*k* = 2), consistency, two-way mixed-effects model. Bold results were considered significant compared to SCD (*p* < 0.01)

## Discussion

In this study, a RCD protocol for TAVR planning on a photon counting detector CT (PCDCT) was evaluated.

**Fig. 3:**
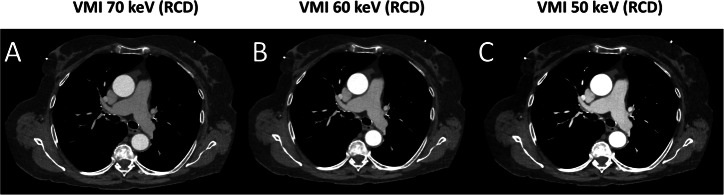
Representative axial images (window width/window level: 700/80 HU) of the same patient acquired with reduced contrast media dose (RCD), combined with virtual monoenergetic images (VMI) at 70 keV (**A**), 60 keV (**B**), and 50 keV (**C**) on a photon-counting detector CT (PCDCT)

**Fig. 4:**
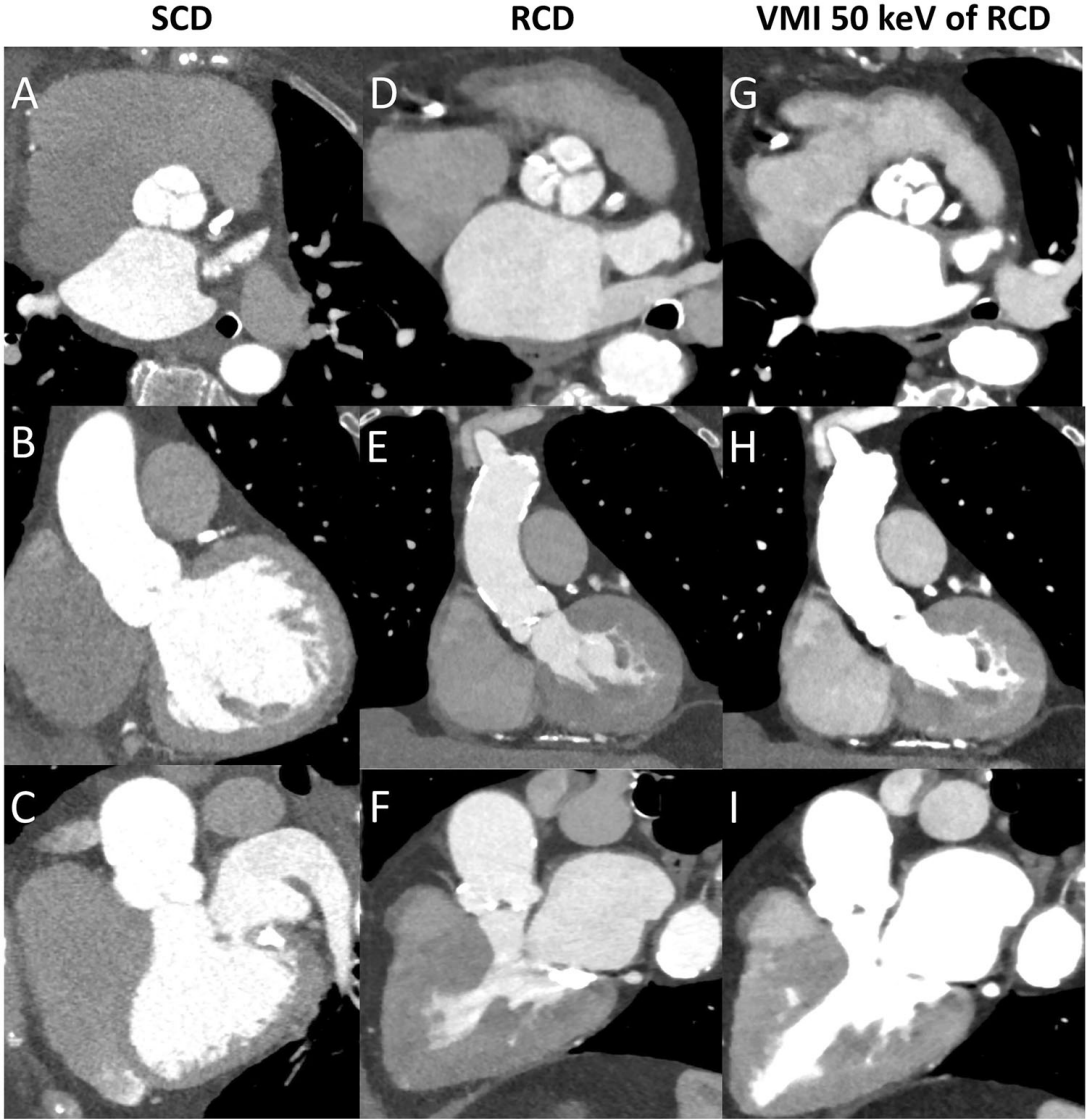
Representative axial, coronal and sagittal images (window width/window level: 700/80 HU) of a patient acquired with standard contrast media dose (SCD) (**A**, **B**, and **C**), a BMI-matched patient with reduced contrast media dose (RCD) (**D**, **E**, and **F**), and corresponding virtual monoenergetic images (VMI) of RCD at 50 keV (**G**, **H**, and **I**) acquired with a photon-counting detector CT (PCDCT)

**Fig. 5:**
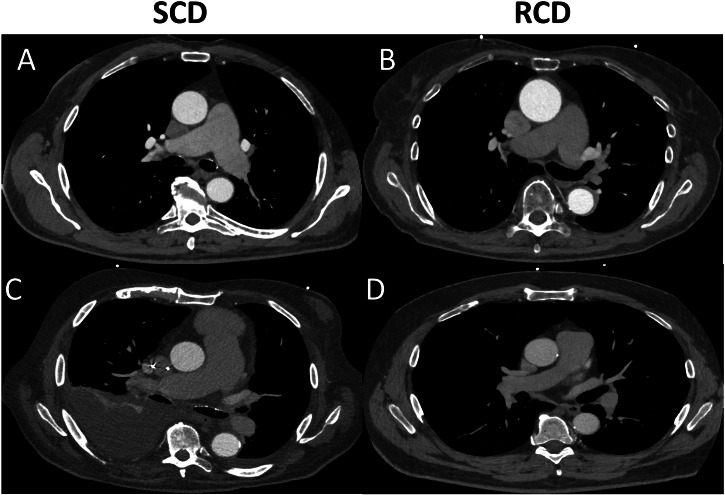
Representative polychromatic axial images (window width/window level: 700/80 HU) of a female (**A**) and a male patient (**C**) acquired with standard contrast media dose (SCD), and BMI-matched female (**B**) and male (**D**) patients with reduced contrast media dose (RCD), acquired with a photon-counting detector CT (PCDCT)

The main findings demonstrate that diagnostic image quality can be maintained with an RCD corresponding to a reduction of 37.5% compared to the standard contrast dose (SCD). Conventional reconstructions (CNs) of RCD scans showed only minor reductions in SNR and CNR compared with standard contrast media protocol (SCD) scans. Importantly, VMI reconstructions of RCD datasets further improved image quality, with VMI at 60 keV and 50 keV restoring—and in some cases exceeding—the SNR and CNR of conventional SCD reconstructions (Figs. 3–5).

PCDCT represents a significant technological innovation in CT imaging, offering enhanced spatial resolution, detailed energy discrimination allowing for improved iodine signal characterization, and noise reduction compared to conventional EIDCT systems [24, 27]. This enables diagnostic imaging with lower contrast media volumes, reducing the risk of contrast-associated nephropathy and improving safety, particularly for patients with pre-existing renal impairment. Furthermore, PCDCT’s improved spatial resolution enables better visualization of fine vascular and parenchymal structures even at lower contrast media concentrations [28, 29]. The current findings confirm and extend previous phantom and clinical studies reporting improved iodine sensitivity and noise performance with PCDCT, and suggest that substantial contrast savings are achievable in routine clinical TAVR imaging [26, 30–32]. In accordance with this, SNR and CNR showed higher values in this study using PCDCT than in comparable studies on DECT [17, 33–35].

Various studies have stated a correlation between contrast-associated nephropathy and contrast media dosage; however, the correlation does not appear to be linear, but rather depends on various factors and is mostly noticed in intra-arterial injection [36–38]. Nevertheless, most studies agree that the lowest possible dose should be aimed for [10, 39]. As mentioned before, there are approaches for a non-contrast assessment of TAVR planning in which, however, only the annulus and calcified plaques can be visualized [3–5].

Previous phantom studies indicate that an even more substantial reduction in contrast media dose could be achieved [40, 41]. However, as the patients were scanned in a clinical setting, the protocol was designed for optimal image quality and contrast media reduction was not maximized. Our study also suggests that further reduction in contrast media dose is feasible.

Recent studies do support the results observed in this study, stating sufficient image quality and preserved SNR and CNR at RCDs for abdominal imaging, CT pulmonary angiography and coronary CT angiography with PCDCT [25, 30, 42–48]. As in previous studies investigating contrast media dose reduction for TAVR using dual-energy CT, there were no limitations in terms of image quality or divergent measurements [34, 49]. In a prospective clinical trial using dual energy CT, contrast media volume could be reduced to 40 mL while maintaining diagnostic image quality, applying low-keV virtual monoenergetic reconstructions. These findings are consistent with the present results and suggest that spectral imaging techniques play a key role in enabling contrast-efficient TAVR imaging. Higashito et al compared CTA of the aorta between PCDCT and EIDCT in a prospective study at matched radiation dose, demonstrating noninferior image quality at reduced contrast media volume [26]. The results of our study do support this conclusion for TAVR planning using a triphasic contrast media protocol, demonstrating a specific application for reducing contrast media in PCDCT.

Recent work proposed a practical rule-of-thumb to adapt contrast media dose in photon-counting CT based on the relationship between photon energy and iodine attenuation [50]. In line with this, in our study, we observed significant improvements in SNR and CNR with low-energy VMI, which can be translated into a further reduction in contrast media volume

There are some limitations to this study. First, the study design is retrospective and single-centered in a relatively small patient cohort. Each patient was scanned only once and matched to a Body Mass Index-equivalent scan of the corresponding cohort. This approach was chosen due to ethical concerns of repeated comparative CT scans with accumulation of non-essential radiation exposure.

Second, only one RCD protocol was tested. In April 2023, we decided to make use of the advantages of PCDCT and created the contrast media-reduced TAVR planning protocol (RCD). In this retrospective study, it was tested against our former standard TAVR planning CT protocol with the use of the photon-counting detector CT (SCD). There are a variety of approaches to creating a sufficient contrast media protocol for TAVR planning. In our institution, we decided to focus on a split-bolus approach followed by a flush of saline solution. Thus, the results may not be generalizable to differing protocols.

In our study, annulus measurements were performed using large field-of-view reconstructions, rather than the dedicated narrow field-of-view reconstructions tailored to the heart and aortic root, although we acknowledge that this approach does not adhere to the clinical standard. However, all measurements were performed in the same reconstructions, and we believe the consistency of the results remains unaffected.

Finally, the contrast media protocol used in this study is fixed, independent of body weight. Body weight–an adapted contrast media application could probably help to further reduce contrast media consumption.

Despite its potential, the widespread adoption of RCD protocols faces challenges. The clinical implementation of PCDCT is still in its early stages, and robust, multi-center studies are needed to validate the generalizability of RCD protocols across diverse patient populations and clinical scenarios. Furthermore, optimizing the trade-off between contrast media dose reduction and image quality for specific applications remains a critical task for further studies.

Looking ahead, the combination of PCDCT with artificial intelligence-based image reconstruction and dose optimization techniques may further refine the reduction of contrast media [51–53]. Such tools could enhance spectral image processing and facilitate automated protocol adjustments tailored to individual patient characteristics. Further potential savings are indicated by the use of low-keV VMI.

## Conclusions

The diagnostic efficacy of transcatheter aortic valve replacement (TAVR) planning assessment using Photon Counting Detector CT (PCDCT) with minimized contrast agent dosing is preserved, presenting a practical approach to conserve contrast media. This can benefit patients with renal impairment, for example, and reduce the negative effects of iodinated contrast media on the environment.

## Data Availability

Data associated with the study will be made available on reasonable request.

## Abbreviations

CI: Confidence interval
CNR: Contrast-to-noise ratio
CR: Conventional reconstruction
HU: Hounsfield Units
ICC: Intraclass correlation coefficient
PCDCT: Photon-counting detector CT
RCD: Reduced contrast media dose
ROI: Region-of-interest
SCD: Standard contrast dose
SNR: Signal-to-noise ratio
SPP: Spectral post processing
TAVR: Transcatheter aortic valve reconstruction
VMI: Virtual monoenergetic images
